# Targeting Breast Cancer: The Familiar, the Emerging, and the Uncharted Territories

**DOI:** 10.3390/biom13091306

**Published:** 2023-08-25

**Authors:** Hamidreza Montazeri Aliabadi, Arthur Manda, Riya Sidgal, Co Chung

**Affiliations:** Department of Biomedical and Pharmaceutical Sciences, Chapman University School of Pharmacy, Harry and Diane Rinker Health Science Campus, Irvine, CA 92618, USA

**Keywords:** breast cancer, targets, signaling pathway, druggable, therapy

## Abstract

Breast cancer became the most diagnosed cancer in the world in 2020. Chemotherapy is still the leading clinical strategy in breast cancer treatment, followed by hormone therapy (mostly used in hormone receptor-positive types). However, with our ever-expanding knowledge of signaling pathways in cancer biology, new molecular targets are identified for potential novel molecularly targeted drugs in breast cancer treatment. While this has resulted in the approval of a few molecularly targeted drugs by the FDA (including drugs targeting immune checkpoints), a wide array of signaling pathways seem to be still underexplored. Also, while combinatorial treatments have become common practice in clinics, the majority of these approaches seem to combine molecularly targeted drugs with chemotherapeutic agents. In this manuscript, we start by analyzing the list of FDA-approved molecularly targeted drugs for breast cancer to evaluate where molecular targeting stands in breast cancer treatment today. We will then provide an overview of other options currently under clinical trial or being investigated in pre-clinical studies.

## 1. Introduction

Cancer is still among the leading causes of morbidity worldwide. In 2020, more than 19 million new cases of cancer were diagnosed, and almost 10 million cancer-caused deaths were reported [1]. In 2020, breast cancer became the most diagnosed cancer in the world, with over 2.3 million new cases and 685,000 in this year [2]. This grim statistic is partly due to the drop in deaths caused by infectious diseases (better sanitation, new antibiotics, and vaccine development) and better control and prevention of cardiovascular diseases [3]. With the explosion of small molecules and antibodies targeting a specific protein involved in the proliferation and/or survival of cancer cells (collectively known as “molecularly targeted drugs”) on top of the expanding repertoire of chemotherapeutic agents, one would think that cancer therapy should not have fallen behind. However, as we all have heard repeatedly, cancer is a heterogenous and plastic disease.

The innate resistance of unresponsive cells is usually explained by tumor heterogeneity. In 2015, Sottoriva et al. proposed a ‘Big Bang’ model of tumor initiation that suggests that after initial oncogenic mutations, future generations acquire further mutations, which are present in discrete populations of cells, leading to spatial heterogeneity [4]. An even more diverse pattern has been observed in other types of cancer. Amir et al. studied two human acute lymphoblastic leukemia samples with viSNE technology and reported a large, irregular population of cells that were more different than similar [5]. Personalized medicine (based on the biomarker expression in each specific patient) has been suggested as a solution to the limited response rate in naïve cells. However, this intra-tumor heterogeneity remains a major hindrance. The sub-population with intrinsic resistance to therapeutic assault would survive and outgrow other cells due to the selection pressure, which promotes relapse and results in an abundance of cells that were once minorities [6]. This “Darwinian clone selection” has been well documented in different types of cancer in response to a variety of molecularly targeted drugs [7].

Efflux proteins (e.g., P-glycoprotein) and their role in acquired resistance to chemotherapeutic agents have been well studied. This desensitization of originally responsive cells has also been observed in response to molecularly targeted drugs [8,9]. This acquired resistance has been reported in different types of cancer (e.g., non-small-cell lung cancer [10,11]) and for different targets (e.g., hormonal therapy in estrogen receptor-positive breast cancer [12] or receptor tyrosine kinases [13,14]). While point mutations are widely accepted mechanisms for this resistance development [15,16,17,18,19], the ability of cancer cells to “switch” to an alternative protein for their survival (known as plasticity) is another important factor. In this case, the initial response to the molecularly targeted drug (as the result of inhibition of the targeted protein) might be diminished due to the overactivation of other proteins and/or signaling axes that could compensate for the loss of function of the inhibited protein [20,21].

Molecularly targeted drugs and the idea of personalized treatment can revolutionize cancer treatment. However, due to the issues explained above, we first need a better understanding of the molecular targets and their potential “crosstalk”. This review focuses on these molecular targets in breast cancer therapy. However, it is important to note other frontline strategies, including surgery and radiotherapy [22]. Breast cancer surgery has historically been the most widely used approach. The radical mastectomy described by Halsted in the late 19th century gave way to the modified Madden mastectomy described in 1972 that preserves both pectoral muscles [23]. A recent narrative literature review reported that local progression-free survival was improved, and distant progression-free survival was worsened by breast cancer surgery with no significant effect on quality of life. It also indicates that the results of surgery at the metastasis site are variable and depend on multiple factors [24]. Also, radiotherapy is an important component of breast cancer management, both locally and at the metastasis site(s). Breast conservation with lumpectomy followed by whole or partial breast radiation is the current standard of care for early breast cancer cases and improves the survival outcome equivalent to mastectomy [25].

## 2. Where We Stand

Table 1 summarizes the molecularly targeted drugs approved by the FDA to be used in breast cancer treatment (as of 10 February 2023), their molecular target, the type of breast cancer for which they are used, and selected ongoing clinical trials for combinational therapies that include each. Out of 23 drugs listed in the table, eight (approximately 35%) target hormone related molecules. There are six drugs (approximately 26%) that target the human HER family of receptors (three of which specifically target HER2). Three FDA-approved drugs in breast cancer treatment (13%) inhibit cyclin-dependent kinase (CDK) 4/6, which regulates cellular transition from the G1 phase of the cell cycle to the S phase [26]. Two drugs on the list (~8.7%) inhibit immune checkpoint pairs, programmed death-1 (PD-1) and programmed death-ligand 1 (PD-L1), that are expressed on T cells and breast cancer cells, respectively. There are also two drugs (~8.7%) that target poly (ADP-ribose) polymerase (PARP) enzymes that are involved in DNA repair. And finally, the last two drugs on the list (~8.7%) target phosphatidylinositol-3-kinase (PI3K) and mammalian target of rapamycin (mTOR), two proteins involved in the PI3K/AKT pathway.

This quick overview reveals a significant emphasis on hormone-focused therapy (in hormone receptor-positive types), followed by HER receptors and CDK. Immune therapy is an emerging field in cancer therapy in general, and PARP inhibitors are an emerging approach in breast, ovarian, and prostate cancers [27]. While targeting the PI3K/AKT pathway does appear on this list, other major signaling pathways are not part of this picture and could be considered uncharted territory in breast cancer treatment.

**Table 1:** List of FDA-approved molecularly targeted drugs in breast cancer treatment and some of the recent clinical trials in combinational therapy using this approach.

Drug	Targeted Protein	Indication	Used in Combination	Selected Combinations in Clinical Trials
Abemaciclib	CDK4/6	HR-Positive, HER2-negative	Fulvestrant, aromatase inhibitors, Tamoxifen	Phase Ib/II (NCT04791384) with elacestrant; Phase II (NCT04432454) with lasofoxifene (ERA); Phase I (NCT05095207) with bicalutamide (AA); Phase I (NCT05464173) with Fulvestrant and chidamide (HDACI); Phase I (NCT03846583) with tucatinib and trastuzumab
Alpelisib	PI3K (predominantly against PI3K catalyst subunit α)	HR-Positive, HER2-negative with PIK3CA mutation	Fulvestrant	Phase I (NCT05143229) with Sacituzumab govitecan; Phase II (NCT05625087) with ribociclib; Phase I (NCT05230810) with tucatinib
Anastrozole	Aromatase	HR-Positive	-	Several with fulvestrant, goserelin, tamoxifen, gefitinib (EGFRI), etc.
Atezolizumab	PD-L1	Advanced or metastatic TNBC	It is only approved in combination with albumin-bound paclitaxel ^†^	Phase II (NCT04759248) with trastuzumab and vinorelbine (chemotherapy); Phase I/II (NCT02708680) with entinostat (HDACI); Phase II (NCT04690855) with Talazoparib; Phase I (NCT04584112) with tiragolumab (TIGIT blocker)
Elacestrant	Estrogen receptor α	ER-positive and HER2-negative with ESR1 mutation	-	Phase III (NCT05512364) with tamoxifen, letrozole, anastrozole, and exemestane; Phase Ib/II (NCT04791384) with abemaciclib; Phase I (NCT05618613) with onapristone (AP)
Everolimus	mTOR	HR-Positive, HER2-negative	Exemestane	Several with letrozole, fulvestrant, goserelin, erlotinib, trastuzumab, etc.
Exemestane	Aromatase	ER-positive	-	Several with Dasatinib (TKI), chidamide (HDACI), raloxifene (ERAA), everolimus, fulvestrant, etc.
Fulvestrant	Estrogen receptor	HR-Positive, HER2-negative	Ribociclib, palbociclib, or abemaciclib (in metastatic cases that got worse with hormone therapy alone)	Several with enzalutamide (second generation AA), neratinib or pyrotinib (TKIs; in HR-positive, HER2-positive cases), goserelin, anastrozole, etc.
Goserelin	LHRH receptor	Advanced breast cancer	-	Phase II (NCT02072512) with fulvestrant and anastrozole; Phase II (NCT00010010) with exemestane; Phase II (NCT00217659) with anastrozole; Phase I (NCT02586675) with tamoxifen and ribociclib
Lapatinib	EGFR/HER2	Advanced HER2-positive	Capecitabine (chemotherapy), or with letrozole (in HR-positive, HER2-positive cases)	Phase I (NCT00424164) with tamoxifen; Phase II/III (NCT03085368) with trastuzumab; Phase II (NCT01272141) with everolimus; Phase II (NCT01275859) with letrozole; and several trials with a variety of chemotherapeutic agents
Letrozole	Aromatase	Early stage or metastatic HR-positive	-	Several with nintedanib (TKI), lenvatinib (TKI), pembrolizumab, palbociclib, anastrozole, ribociclib, tucatinib, dalpiciclib (CDK4/6 inhibitor), bevacizumab (VEGF inhibitor), etc.
Margetuximab	HER2	Metastatic HER2-positive	-	Phase II (NCT04262804) and Phase III (NCT02492711) with trastuzumab; Phase II (NCT04425018) with pertuzumab; Phase I (NCT03219268) with tebotelimab (PD-1 and LAG-3 blocker)
Neratinib	EGFR, HER2, HER4	HER2-positive	Capecitabin (chemotherapy) in advanced or metastatic cases	Phase II (NCT03289039) with Fulvestrant; Phase II (NCT01111825) with temsirolimus (mTOR inhibitor); Phase II (NCT00915018) with trastuzumab)
Olaparib	PARP enzymes	HER2-negative with mutations in BRCA1 or BRCA2	-	Phase II (NCT03025035) with pembrolizumab; Phase II (NCT03594396) with durvalumab (PD-L1 antibody); Phase II (NCT05536128) with fulvestrant; Phase II (NCT02849496) with atezolizumab
Palbociclib	CDK4/6	HR-Positive, HER2-negative	Fulvestrant, aromatase inhibitors	Several with letrozole, avelumab (PD-1 antibody), erdafitinib (FGFR inhibitor), inavolisib (PI3Kα inhibitor), etc.
Pembrolizumab	PD-1	TNBC	Chemotherapy	Several with tamoxifen, mifepristone (PRA), fulvestrant, lenvatinib (TKI), olaparib, exemestane, trastuzumab, etc.
Pertuzumab	HER2 (and EGFR, HER3 and HER4)	HER2-positive	Trastuzumab	Phase II (NCT05574881) with dalpiciclib (CDK4/6 inhibitor) and fulvestrant; Phase II (NCT03988036) with pembrolizumab; Phase III (NCT05132582) with tucatinib; Phase II (NCT03820141) with durvalumab (PD-L1 antibody)
Ribociclib	CDK4/6	HR-Positive, HER2-negative	Fulvestrant, aromatase inhibitors	Phase Ib/II (NCT02657343) with trastuzumab; Phase II (NCT05625087) with Alpelisib; Phase I (NCT02586675) with tamoxifen; Phase I (NCT01857193) with everolimus/exemestane
Talazoparib	PARP enzymes	HER2-negative with mutations in BRCA1 or BRCA2	-	Phase I/II (NCT03964532) with avelumab (PD-1 antibody); Phase II (NCT04690855) with atezolizumab; Phase I/II (NCT05035745) with Selinexor (XPO1 inhibitor)
Tamoxifen	Estrogen receptor	Metastatic breast cancer	Surgery and radiotherapy	Several with pembrolizumab, gefitinib (EGFRI), raloxifene (ERAA), toremifene, anastrozole, etc.
Toremifene	Estrogen receptor	Metastatic breast cancer (ER-positive or unknown)	-	Phase IV (NCT02344940) with tamoxifen; Phase IV (NCT02089854) with anastrozole; Phase III (NCT02132390) with goserelin; Phase III (NCT00044291) with atamestane (AI)
Trastuzumab	HER2	HER2-positive	Chemotherapy in hormone receptor negative, high-risk, or metastatic breast cancer	Several with pertuzumab, bevacizumab (VEGF inhibitor), everolimus, monalizumab (NKG2A inhibitor), fulvestrant/pertuzumab/dalpiciclib (CDK4/6 inhibitor), and several chemotherapies
Tucatinib	HER2	HER2-positive	Trastuzumab and capecitabine ^††^	Phase I/II (NCT05230810) with alpelisib

AA: Anti-androgen; AI: Aromatase inhibitor; AP: Anti-progesterone; BRCA: Breast cancer gene; CDK: Cyclin-dependent kinase; EGFRI: Epidermal growth factor receptor inhibitor; ER: Estrogen receptor; ERA: Estrogen receptor antagonist; ERAA: Estrogen receptor agonist/antagonist; ESR1: Estrogen receptor 1; FGFR: Fibroblast growth factor receptor; HDACI: histone deacetylase inhibitor; HER: Human epidermal growth factor receptor; HR: Hormone receptor; LAG-3: Lymphocyte-activating protein; LHRH: Luteinizing hormone releasing hormone; mTOR: Mammalian target of rapamycin; PARP: Poly (ADP-ribose) polymerase; PD-1: Programmed Death-1; PD-L1: Programmed death-ligand 1; PI3K: Phosphatidylinositol-3-kinase; PRI: Progesterone receptor antagonist; TIGIT: T cell immunoreceptor with Ig and ITIM domains; TKI: Tyrosine kinase inhibitor); TNBC: Triple negative breast cancer; VEGF: Vascular Endothelial Growth Factor; XPO1: Exportin 1 (nuclear transport protein). Megestrol acetate was not included in this table since it is used as a cytotoxic agent in breast cancer. The information included in this table was extracted from the NIH list of FDA-approved drugs for breast cancer treatment (https://www.cancer.gov/about-cancer/treatment/drugs/breast; updated on 10 February 2023) and https://clinicaltrials.gov (updated 8 February 2023). ^†^ The manufacturer of atezolizumab, Genentech (under brand name Tecentriq), announced that it is “voluntarily withdrawing the breast cancer indication” for this drug in the United States [28]. ^††^ In patients who have received at least one HER2 inhibitor and breast cancer has metastasized.

## 3. The Familiar

### 3.1. Hormone Therapy

Tamoxifen was first synthesized as a contraceptive in 1962. The project failed since this molecule surprisingly stimulated ovulation. In the 1980s, clinical trials showed its efficiency in breast cancer when used with chemotherapy. Consequent trials showed the efficiency of tamoxifen in the prevention of the development or re-appearance of breast cancer, and a new approach to breast cancer therapy was born [29]. Hormone therapy still takes up a big share of the FDA-approved drugs for breast cancer therapy, and it is not surprising since the majority of the diagnosed breast cancers are estrogen receptor (ER) positive (~80%) or ER and progesterone receptor (PR) positive (~65%) [30,31]. Among eight FDA-approved drugs categorized as hormone therapy, there are three aromatase inhibitors that lower estrogen levels, three selective estrogen receptor modulators (SERMs), one elective estrogen receptor downregulator (SERD; fulvestrant) that destroys the receptor, and one luteinizing hormone-releasing hormone (LHRH) agonist that, with chronic use, reduces estradiol levels. Steroid hormones play a role in carcinogenesis in breast cancer and would result in enhanced cell growth, development, differentiation, and homeostasis [32].

**Figure 1:**
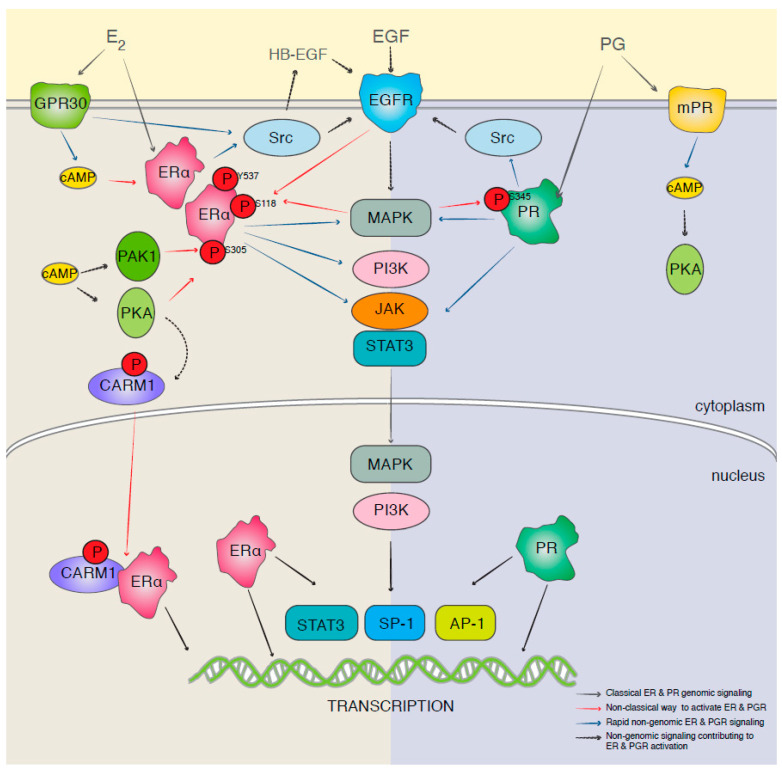
A schematic presentation of the genomic and nongenomic estrogen and progesterone receptors signaling and crosstalk (reproduced with permission from [36]).

However, there is much more to the estrogen/progesterone receptors and their signaling than meets the eye at first glance. The effect of estrogen is mainly exerted through estrogen receptor alpha (ERa) and/or estrogen receptor beta (ERb) [33]. Inactive Era binds to heat-shock proteins in the cytoplasm. In the presence of estrogen, it dissociates and binds to estradiol, dimerizes, enters the nucleus, and binds to estrogen response element (ERE) to act as a transcription factor to enhance expression of Cyclin D1, which activates CDK4/6 [34]. It also increases the expression of the mouse double minute 2 homolog (MDM2), which promotes cell survival via different mechanisms, including suppression of p53 [35]. However, estrogen and progesterone signaling pathways have a non-genomic pathway as well, which includes crosstalk with growth factor receptors and G-protein-coupled (GPR) receptors [36]. It has been reported that estrogen transactivates EGFR via GPR30, which in turn activates the Ras/Raf pathway and its downstream effectors (MEK and ERK-1/-2) [37]. It has also been shown that PR could activate Src-MAPK and AKT pathways [38]. Some of the crosstalk between estrogen and other major signaling pathways is demonstrated in Figure 1.

### 3.2. HER Family of Receptors

**Figure 2:**
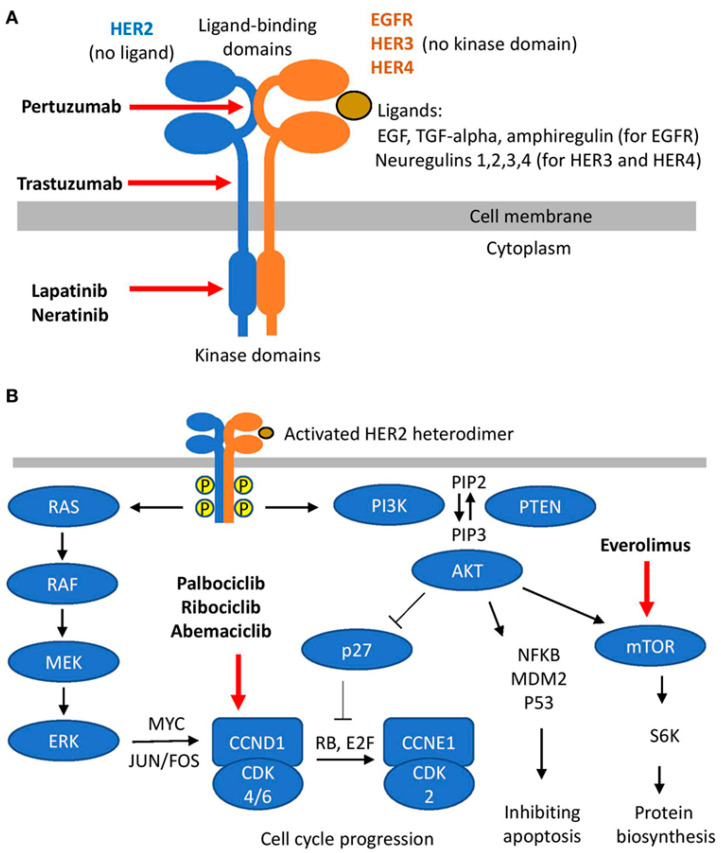
A schematic presentation of HER family signaling; (**A**) heterodimer formation, ligands, and HER2 targeting molecules; and (**B**) downstream signaling cascades triggered by heterodimers (reproduced with permission from [42]).

Targeting the family of HER receptors (especially HER1, EGFR, and HER2) seems like a logical approach in HER2-positive breast cancer, which makes up approximately 30% of breast cancer cases [39]. However, the signaling cascades that are triggered by this family of receptor tyrosine kinases (RTKs) involve a wide array of proteins and intracellular mechanisms, including the same mechanisms that are activated by estrogen. The function of HER2 is dependent on dimerization with another HER2 (homodimerization) or other members of the family (heterodimerization) [40]. This dimerization is the trigger for the activation of a variety of proteins, enzymes, messengers, and transcription factors that induce an array of responses involved in the carcinogenesis of the mammary gland [41]. Unlike other members of the receptor family, HER2 does not have a ligand (Figure 2; [42]) and is reported to stabilize and enhance dimerization [41]. The dimerization mainly activates the Ras/Raf/MEK/ERK and PI3K/AKT pathways, which increase the expression of Cyclin D1 and activate CDK4/6 directly and indirectly, respectively [42,43,44]. There is also evidence for activation of the JAK/STAT pathway by HER2 and EGFR homo/hetero dimers, which would lead to enhanced expression of proteins that would activate epithelial-to-mesenchymal transition (EMT), which increases the risk of metastasis [45,46].

The ties between the estrogen receptor and RTKs are easy to detect. While different receptors are involved in the initiation of the pathways, there are common downstream effectors and mechanisms. The involvement of major signaling pathways in estrogen and HER2 signaling and activation of CDK4/6 by both routes were mentioned before. In fact, crosstalk between the two signaling cascades and the role of EGFR, HER2, and insulin-like growth factor-1 receptor (IGF1R) in resistance to hormone therapy have been reported [43]. Another common effector is the steroid receptor activator (SRC). Although many details are still unknown about the role of c-SRC, its role in the development and progression of different types of cancer is well established [47]. Interaction of c-SRC with the estrogen receptor leads to activation of the downstream effectors, and reports indicate a role for c-SRC in resistance to hormone therapy [48,49]. Interestingly, targeting c-SRC has been reported to be effective in overcoming resistance to trastuzumab, one of the FDA-approved HER2 targeting drugs in breast cancer treatment [50]. We will discuss SRC in more detail later in the manuscript. A wide array of other HER2 inhibitors is in clinical trials (including neratinib) [51,52,53].

### 3.3. CDK4/6

As mentioned before, three drugs targeting CDK4/6 have been approved by the FDA to be used in HR-positive and HER2-negative breast cancers. CDK4 and CDK6 are members of a family of serine/threonine kinases that are activated by binding to cyclins (mostly cyclin D in breast cancer) and enhance cell cycle progression and cell proliferation [54]. The CDK4/6 complex is an important mediator for transition into S phase and plays an important role in carcinogenesis and the progression of breast cancer [55]. On the other hand, resistance to HER2 targeting molecules has encouraged studies of the effect of CDK4/6 inhibitors in HER2-positive cases that show resistance to HER2 inhibitors [56,57]. It is important to note that CDK4/6 is considered a downstream effector for both estrogen- and RTK-triggered pathways. Many additional first- and second-generation CDK4/6 inhibitors are in clinical trials and have been reviewed recently in multiple publications [58,59,60]. Despite promising results, resistance to CDK4/6 inhibitors also seems to be inevitable and seems to involve a variety of mechanisms, including abnormal activation of CDK4/6, loss of retinoblastoma protein (pRb), cyclin E activation, loss of PTEN, and activation of alternative pathways including Ras, fibroblast growth factor receptor 1 (FGFR1), and/or PI3K/AKT pathways [61,62].

## 4. The Emerging

### 4.1. Immune Checkpoint Inhibitors

**Figure 3:**
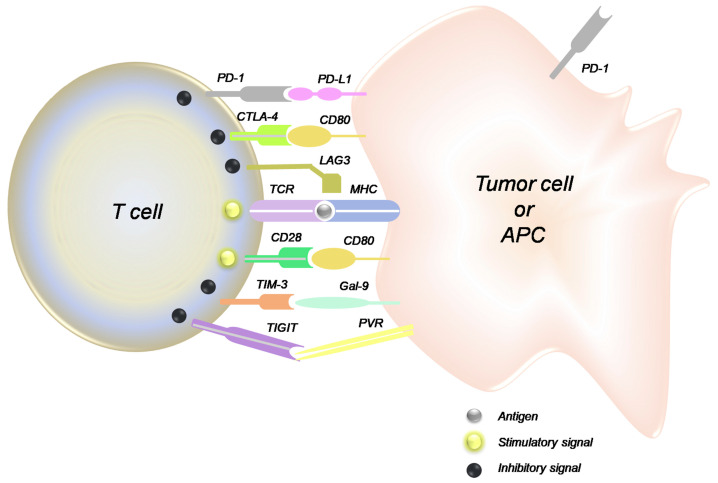
Interactions among the immune checkpoints on tumor cells and the tumor-specific T cell (reproduced with permission from [65]).

Inherent genetic and epigenetic changes in cancer cells create a wide variety of antigens, and therefore, tumor cells develop immune resistance mechanisms, including immune checkpoints [63]. Immunotherapy has been a significant recent advancement in cancer treatment, and while breast cancer did not initially seem to be a great candidate for this approach (due to low immunogenicity), immune checkpoint inhibitors have found their way into breast cancer therapy [64]. Inhibition of immune checkpoints can be achieved via two general approaches: targeting the ligand expressed on the cancer cell or the inhibitory receptor on the cancer-specific T cells. Both approaches have been investigated, and each presents different opportunities and challenges. Among the targets in this category, the PD-1/PD-L1 pair has been explored more extensively, and both FDA-approved drugs in this category for breast cancer therapy target this pair. The other pairs that trigger an inhibitory signal for immune response to cancer cells are CD80/CTLA-4, MHC/LAG3, Gal9/TIM3, and PVR/TIGIT (Figure 3).

The recent clinical trials performed on immune checkpoint inhibitors (other than the two drugs already approved by the FDA for breast cancer) are summarized in Table 2. As expected, targeting PD-1/PD-L1 makes up the majority of the clinical studies in the pipeline. This could at least partially contribute to the chronological order of the discovery of these targets, with PD-1 being discovered in 1992 [66]. More interestingly, among the clinical trials focused on PD-1/PD-L1, most of the studies target PD-1 (expressed on the T cells). This could be explained (again, at least partially) by the fact that T cells are more accessible than solid tumor cells. It is also noticeable that most of the combinations studied in clinics are focused on targeting VEGF/VEGFR or double-targeting immune checkpoints (durvalumab and tremelimumab; nivolumab and ipilimumab; or spartalizumab and LAG525). On the other hand, CTLA-4 was first discovered in 1987 [67] (earlier than PD-1), but it was not until 1996 that anti-CTLA-4 antibodies were reported to reduce the rate of tumor growth [68]. Keeping up with the same trend, however, targeting CTLA-4 (expressed on T cells) seems to be the focus of the CD80/CTLA-4 pair, as no CD80-targeting drug seems to be coming up in the pipeline. The only molecule targeting LAG3 studied in breast cancer at the moment is LAG525 (Ieramilimab), which has been studied in combination with a PD-1 inhibitor (Spartalizumab) [69]. Also, the TIM-3 inhibitor Sabatolimab has entered clinical trials in combination with Spartalizumab [70].

### 4.2. PARP Inhibitors

At first glance, poly (ADP-ribose) polymerase (PARP) inhibitors might seem to work like chemotherapeutic agents since they prevent DNA repair. However, this category of drugs has a very specific molecular target: PARP enzymes. Inherited mutations in breast cancer 1 and 2 (BRCA1 and 2) are detected in 11–20% of TNBC and 5–7% of all types of breast cancer cases and are the most common hereditary defect in breast cancer [89]. These mutations result in the loss of function of the genome-protecting “tumor suppressor” proteins and increase the risk of DNA damage [90]. PARP1 and 2 are involved in base excision repair (BER), and PARP1 is involved in nucleotide excision repair (NER), both of which processes enable DNA repair. Therefore, PARP inhibitors cause genomic instability and cell cycle arrest in cells with damaged DNA and are approved for clinical use in HER2-negative breast cancer with BRCA1 and 2 mutations [91], even though many reports indicate the potential for the efficiency of this category of drugs in TNBC with BRCA1/2 mutations [89,92,93,94].

Many other PARP inhibitors are in different stages of clinical trials. Fluzoparib (a PARP1 inhibitor) has been investigated in combination with camrelizumab (a PD-1 inhibitor) and apatinib (a VEGFR inhibitor) in TNBC (NCT03945604, phase I) with signs of efficiency, especially in patients with BRCA 1/2 mutations [83]; in combination with dalpiciclib (a CDK4/6 inhibitor) in HR-positive, HER2-negative breast cancer (NCT05759546, phase II); with dalpiciclib and fulvestrant in metastatic breast cancer (NCT05759546, phase II); with camrelizumab and Nab-paclitaxel in HER2-negative breast cancer with homologous recombination repair (HRR) gene mutation (NCT05761470, phase II); and with apatinib (NCT04296370, phase III) in HER2-negative breast cancer with BRCA mutations. Niraparib (a PARP 1/2 inhibitor) has shown signs of efficiency in HER2-negative breast cancer with a BRCA mutation (NCT01905592, phase III) when used alone compared to chemotherapy [95]. A phase II clinical trial (NCT00540358) has shown that iniparib (another PARP1 inhibitor) improves clinical outcome in metastatic TNBC when added to chemotherapy [96]. Interestingly, we failed to find any more recent clinical trials on this drug. A recent Phase I study on veliparib (a PARP 1/2 inhibitor) alone (NCT00892736) has shown signs of efficiency in TNBC patients (even without a BRCA mutation) [97]. Many other clinical trials have also been performed on veliparib in different combinations. Overall, it seems that clinical research on PARP inhibitors is ongoing, and the benefits of this approach might not be limited to HER2-negative breast cancer with BRCA1 and 2 mutations.

### 4.3. PI3K/AKT Pathway Inhibitors

Alpelisib (targeting the PI3K catalyst subunit α or PI3KCA) and everolimus (targeting mTOR) are already approved by the FDA for the treatment of HR-positive, HER2-negative breast cancers (with a PIK3CA mutation for alpelisib). The PI3K/AKT pathway is the most abnormally activated pathway in breast cancer [98] and one of the most complicated signaling axes, with many crosstalks with other major signaling pathways. It is mainly activated via the HER family of receptors; however, activation via the G protein-coupled receptors (GPCRs), cytokine receptors, and integrins is also reported [99]. This triggers the phosphorylation of the serine–threonine kinase AKT by phosphoinositide-dependent protein kinase 1 (PDK1). PTEN is an important inhibitor of PI3K activation [99,100]. Activation of AKT in turn triggers mTOR complexation, which results in mTOR complexes 1 and 2 (mTORC1 and mTORC2). mTORC2 enhances the activity of AKT through positive feedback [101]. AKT has a wide range of downstream proteins and prevents apoptosis via inhibition of Forkhead (FoxO) and bcl2-antagonist of cell death (BAD) [102]; cell growth (via mTORC1 and activation of S6K and eIF4E [103]); and cell cycle (through cyclin D and CDK4/6 [104,105]). Therefore, many other proteins can potentially be molecular targets in the inhibition of the PI3K/AKT pathway. A rather complicated downstream transcription factor linked to PI3K/AKT pathways is the nuclear factor Kappa B (NF-κB). NF-κB is mostly known as a pro-inflammatory transcription factor; however, it is also involved in the regulation of the expression of multiple proteins involved in tumorigenesis, apoptosis, cell cycle progression, resistance, and metastasis in many types of cancer, including breast cancer [106]. It is activated via canonical (via tumor necrosis factor receptor, or TNFR) and noncanonical (via CD40) pathways [107]. However, many studies have pointed out the activation of NF-κB by the PI3K-AKT pathway [106,107,108,109,110].

Many studies have shown the role of the PI3K/AKT pathway in triple-negative breast cancer (TNBC) and HER2-positive breast cancer. As mentioned before, HER2 is one of the activators of PI3K [102], and therefore, targeting the PI3K/AKT pathway seems to be a viable avenue to explore in HER2-positive breast cancer. This signaling pathway could also be deregulated in TNBC [111], which could be due to activating mutations in PI3KCA or loss of function of PTEN and/or proline-rich inositol polyphosphatase (PIPP) [112]. Also, activation of other signaling pathways (e.g., BRAF) could contribute to overactivation of the PI3K/AKT pathway [113]. On the other hand, there are combined mutations (e.g., PI3KC and AKT1) that could occur in up to 30% of advanced TNBCs [114]. It has also been shown that everolimus is equally active in PIK3CA wild-type and mutant types of breast cancer, which suggests that other pathways (e.g., MAPK signaling) could activate mTOR complexes even in the absence of PI3K overactivation [114]. Many recent reviews have been published about the development of new pan-PI3K inhibitors, PI3K isoform-specific inhibitors, AKT inhibitors, mTOR inhibitors, and dual inhibitors (e.g., mTORC1/C2 inhibitors or PI3K/mTOR inhibitors) for treatment of TNBC [98,112,114,115,116,117]. Table 3 summarizes some of the most recent clinical trials for drugs targeting this pathway (specifically in HER2-positive breast cancer, or TNBC). Among the combinatorial approaches in trials, exploring the combination of an AKT inhibitor (Uprosertib) and an MEK1/2 inhibitor (trametinib) seems to be an innovative cross-pathway approach [118].

## 5. The Uncharted

### 5.1. Src

**Figure 4:**
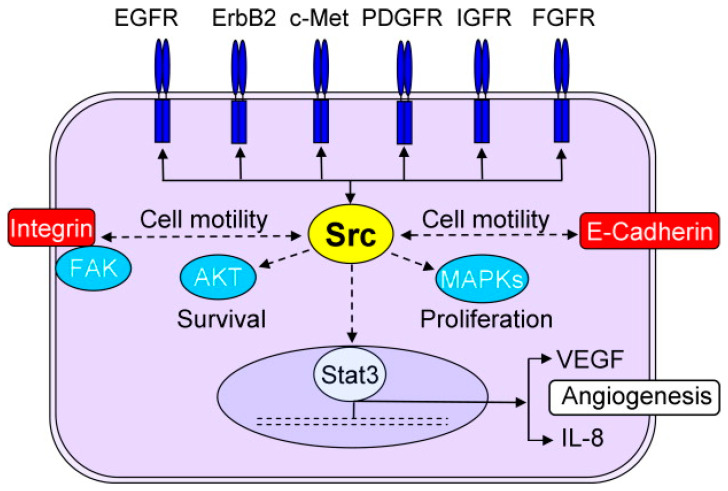
The central role of Src in several mechanisms involved in progression of breast cancer (reproduced with permission from [125].

While the Src family of kinases has been long identified and investigated as a protein involved in breast cancer and hyperactivated in many types of cancer, it has just recently become an attractive target for molecularly targeted drugs due to gaps in knowledge in its role and activation mechanisms [47]. The role of these non-receptor kinases has been indicated in a variety of mechanisms involved in the progression of breast cancer (including metastasis and resistance) [121]. Src is activated by a wide array of membrane receptors (including EGFR and HER2); however, it is also reported to provide positive feedback for activation of these receptors (Figure 4), which means that Src activation does not necessarily require HER2 signaling [39]. In fact, a crucial role has been reported for Src in resistance to HER2 inhibitors, including trastuzumab [50] and lapatinib [122]. Src also has an inhibitory effect on PTEN [123] and plays a role in prolactin signaling [124]. Additionally, Src has interactions with integrin and E-cadherin and crosstalks with all three major signaling pathways in cancer cells, which makes it a candidate for the title of “nodal” protein.

However, a quick look at the recent clinical trials involving Src inhibitors explains the lack of any drugs in this category among the breast cancer treatment plans (Table 4). The only promising result among those efforts is surprisingly reported for non-combinatorial use of bosutinib, with some progression-free and overall survival rates. It is noteworthy that most of the drugs categorized as Src inhibitors are in fact general kinase inhibitors with different IC50s for different kinases (including Abl, AXL, and EGFR). Many other non-specific Src inhibitors (e.g., nintedanib as mostly an angiokinase inhibitor, pelitinib as mostly an EGFR inhibitor, ponatinib as mostly an Abl inhibitor, and resveratrol). There are also many investigational Src inhibitors (some with more specificity towards Src, including PP1 [126] and PP2 [127], SU6656, KX1-004 [128], eCF506, DGY-06-116 [129], and Src inhibitor-1 [130]) that have not reached clinical trials in breast cancer yet. While inhibition of Src in breast cancer has not produced the expected results yet, more discoveries about this important protein in the future can change this bleak overview. 

### 5.2. RAS/RAF/MEK/ERK Pathway

**Figure 5:**
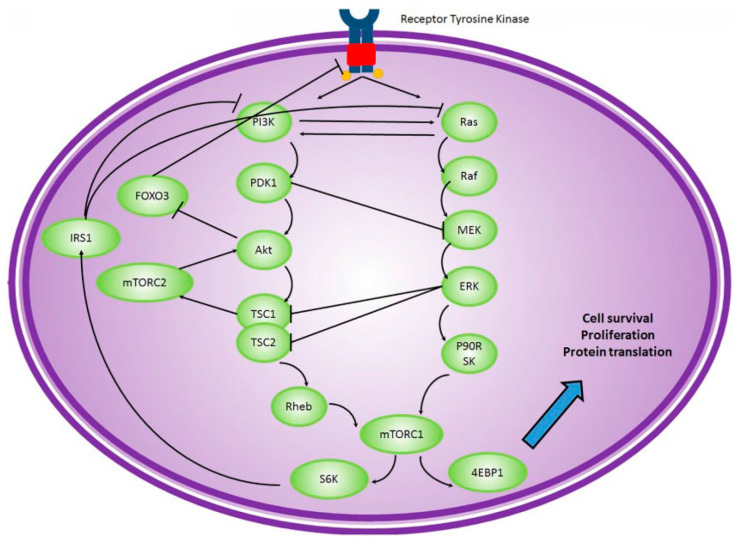
The crosstalk between Ras/Raf/MEK/ERK and PI3K/Akt signaling pathways (reproduced with permission from [141]).

Mutations in the RAS/RAF/MEK/ERK pathway are not as common in breast cancer; however, overactivation of this pathway is common [132], which could be due to extensive crosstalk with PI3K/AKT (Figure 5) and other major signaling pathways. This also indicates an important role for this pathway in the progression of breast cancer and the possibility of identification of the novel molecular targets for treatment. Among Ras-targeting drugs (e.g., ARS-853, ARS-1620, AMG510 (sotorasib), MRTX849, sesquiterpene, and TAN1813 [133,134]), none have entered clinical trials yet. However, sotorasib is approved by the FDA to be used in metastatic non-small cell lung cancer (NSCLC), and sesquiterpene has shown efficiency in MDR breast cancer cells in vitro [135]. Targeting Raf in breast cancer has been more extensively studied in clinics. While vemurafenib is approved by the FDA for metastatic melanoma and a rare type of blood cancer known as Erdheim–Chester disease, none of the three clinical trials on clinicaltrials.gov have reported results. However, a 2020 Phase IIa trial in patients with advanced salivary gland carcinoma showed an objective response in a patient with a Braf mutation [136]. Regorafenib is another Raf inhibitor in multiple clinical studies, but there is no available data at this point. On the other hand, sorafenib (with FDA approval for inoperable hepatocellular carcinoma that also inhibits VEGF) has been studied in more than 30 clinical trials, many of which have reported results. The combination of sorafenib and vinorelbine (a vinca alkaloid) has shown promising results in metastatic breast cancer patients in a phase I/II study, associated with some toxic effects that necessitated dose reduction [137]. However, a 2017 phase II study in HER2-negative metastatic breast cancer patients did not show a positive impact on progression-free survival for paclitaxel/sorafenib in comparison to paclitaxel alone [138]. In other phase II and III studies, addition of sorafenib to docetaxel [139] or capecitabine (a prodrug for fluorouracil) [140], respectively, in advanced or metastatic HER2-negative breast cancer patients did not improve the effect of chemotherapy and only increased the rate of toxicity. The failure of this type of study could potentially be related to a lack of HER2 expression in the selected patient population.

Inhibition of mitogen-activated protein kinase (MEK; also known as MAPKK) has also been extensively studied. Among MEK inhibitors, trametinib is probably the first drug to enter clinical trials involving breast cancer patients. In 2013, a phase 1b study in patients with advanced solid tumors (including breast cancer) showed an increase in myelosuppression of gemcitabine when trametinib was added to treatment, but other toxicities were reported as “manageable” [142]. A phase II study in 2020 conducted on solid tumors and lymphomas with non-V600 BRAF mutations did not show promising results for trametinib (out of 32 patients, only one with breast ductal adenocarcinoma and a BRAF G469E mutation showed a partial response) [143]. And finally, a phase I study on the combination of Uprosertib (an AKT inhibitor) and trametinib in patients with solid tumors did not show significant efficiency and was not well tolerated by the patients either [118]. Among other MEK inhibitors, selumetinib did not improve the outcome of treatment after addition to fulvestrant [144], and recently, cobimetinib increased the percentage of progression-free survival and objective response rate in metastatic TNBC after addition to paclitaxel, which was not statistically significant [145]. Another MEK inhibitor widely studied in clinics is binimetinib; however, all the studies in breast cancer patients have yet to report results. Many molecules targeting ERK and downstream proteins (including MYC, C-fos, and C-Jun) have been developed and studied; however, no clinical data is available at this time. Overall, the lack of clinical studies on inhibitors of this signaling pathway in HER2-positive breast cancer is noteworthy.

### 5.3. JAK/STAT Pathway

The JAK/STAT pathway is another major signaling pathway in cancer cells, and we have recently reviewed its importance in cancer progression [46]. While there are multiple JAK inhibitors that have been globally approved for clinical use, none are for breast cancer treatment. Among these drugs, ruxolitinib is the most studied JAK inhibitor in clinical trials involving breast cancer patients. The most recent clinical study on JAK inhibitors in breast cancer was reported in 2021 on trastuzumab-resistant HER2-positive breast cancer patients, and the addition of ruxolitinib to trastuzumab did not improve the treatment outcome in these patients [146]. In another study in the same year, the combination of ruxolitinib with paclitaxel showed promising results in HER2-negative metastatic breast cancer patients [147]. However, a previous phase II study on ruxolitinib single therapy in metastatic TNBC had not shown significant efficacy [148]. On the other hand, none of the few clinical studies involving STAT inhibitors in breast cancer treatment have reported any results at this point. Therefore, despite extensive data indicating a pivotal role for the JAK/STAT pathway and the extensive crosstalk with other signaling axes in cancer, targeting components of the JAK/STAT pathway has yet to provide expected results.

### 5.4. Others

#### 5.4.1. PPAR

Peroxisome proliferator-activated receptors (PPARs) are a family of nuclear receptors that are involved in lipid and energy homeostasis and include PPARα, PPARγ, and PPARβ/δ [149]. There are reports indicating the effect of PPARα in the regulation of the NF-κB and PI3K/AKT pathways, and PPARα agonists have been studied in combination with other molecularly targeted drugs, chemotherapy, and radiotherapy [150]. A phase I study completed in 2022 investigated the use of TPST-1120 alone and in combination with nivolumab (targeting PD-1) in different advanced cancers, including TNBC; however, the results of the study have not been reported yet.

#### 5.4.2. Syndecans (SDCs)

Syndecans (SDCs) are cell surface heparan sulfate proteoglycans and play a role in many cellular mechanisms, including proliferation [151]. The sulfate chains in SDCs act as binding sites for other proteins, growth factors, and matrix molecules, allowing SDCs to mediate specific types of cell–cell and cell–matrix interactions. These interactions affect cellular functions including cell signaling, migration, adhesion, proliferation, and differentiation [152]. Extensive crosstalk has been reported for SDCs and nuclear hormone receptors, estrogen and progesterone receptors, and PPARγ [153]. Abnormalities in the expression or function of SDCs can lead to poor prognosis and aggression in breast cancer. SDCs and heparanase (an enzyme that cleaves heparan sulfate) have been studied as potential targets in cancer treatment [154]. Heparanase inhibitors have also been developed as drugs and vaccines to be used in cancer treatment. PG545 (a synthetic heparan sulphate mimetic that decreases heparanase expression) created by Progen Pharmaceuticals has been shown to suppress tumor growth in MDA-MB-231 xenografts and inhibit angiogenesis in animal studies [155]. PI-88 is another heparanase inhibitor used in animal models that reduced tumor growth in 13762 MAT cells by inhibiting metastasis and tumoral angiogenesis [156]. Monoclonal antibodies (including the antibody/drug conjugate Indatuximab Ravtansine) have also shown promising results in TNBC patient-derived xenograft models [157].

#### 5.4.3. RUNX2 and HDACs

RUNX2 is a transcription factor involved in the differentiation of human osteoblasts; it is activated by multiple signaling pathways, including PI3K/AKT and NF-κB, and plays a role in bone metastasis of different cancer types, including breast cancer [158]. RUNX2 modifies multiple pathways and can indirectly be involved in angiogenesis, cancer metastasis, proliferation, and drug resistance [159]. One significant cofactor that can regulate RUNX2 expression is histone deacetylase (HDAC) [160]. Currently, multiple HDAC inhibitors are under investigation in clinical trials. Vorinostat, entiniostat, and panobinostat are among the most studied HDAC inhibitors in clinical trials, which date back to as early as 2001. The reports in the literature started with vorinostat in relapsed or refractory breast cancers, which did not show any positive response due to “limited drug exposure” [161]. However, another monotherapy phase II study in the same year showed a relatively positive response in metastatic breast cancer [162]. A 2011 phase II study combining vorinostat with tamoxifen showed some activity in hormone therapy-resistant breast cancer and indicated that HDAC2 expression levels could be a predictive marker for the success of this approach [163]. Another promising combinatorial approach was reported in a phase Ib study with vorinostat and ixabepilone in metastatic breast cancer [164]. The combination of vorinostat and an aromatase inhibitor in ER-positive, HERS-negative metastatic breast cancer resistant to aromatase inhibitors showed promising results in a Phase II study reported in 2020 [165].

There are also several reports on clinical trials involving entinostat in breast cancer. In a phase II study reported in 2013, the combination of entinostat with exemestane in ER-positive metastatic breast cancer patients with resistance to exemestane therapy showed promising results compared to exemestane alone [166]. However, a phase III study reported in 2021 did not show any improvement in survival for the exemestane/entinostat combination in aromatase inhibitor-resistant advanced HR-positive, HER2-negative breast cancer patients [167]. Interestingly, in a separate study reported in 2023, the exemestane/entinostat combination showed significant improvement in progression-free survival compared to exemestane in Chinese patients with HR-positive advanced breast cancer that relapsed/progressed after endocrine therapy [168]. Another Phase II study published in 2016 showed that the combination of 5-azacitidine and entinostat was well tolerated in advanced hormone-resistant or TNBC patients; however, the primary endpoint of the study was not met [169]. And finally, despite a few completed clinical trials with results (NCT00567879, NCT00777335, and NCT00777049) involving panobinostat, the only published report goes back to 2016 for a phase I study on letrozole/panobinostat in metastatic breast cancer patients that showed partial response in patients receiving a higher dose of 30 mg of panobinostat three times a week [170].

#### 5.4.4. Hyaluronic Acid

Hyaluronic acid (HA) is a naturally occurring polymer and a glycosaminoglycan component of the extracellular matrix [171]. Overexpression of HA could indicate a poor prognosis, as it could be involved in progression, metastasis, and multidrug resistance [172]. A meta-analysis of breast cancer cases shows a correlation between high HA levels and lower disease-free survival, recurrence-free survival, and progression-free survival rates [173]. Cluster of differentiation 44 (CD44) is the main HA receptor and is also one of the markers for cancer stem cells [174]. The CD44 pathway plays a role in the motility and migration of cancer cells, which could lead to metastasis [175]. Hyaluronic acid has also been used as a surface moiety on nanoparticles used in drug delivery due to its biocompatibility, biodegradability, and ease of cellular internalization [176]. Bivatuzumab is a monoclonal antibody for CD44 V6, and a conjugation of bivatuzumab and the cytotoxic mertansine has been investigated in a phase I study in pre-treated metastatic breast cancer, which showed efficacy in stabilizing the patient [177]. However, the study was discontinued, and this approach does not seem to be being pursued any further.

#### 5.4.5. NDRG1

N-Myc downstream-regulated gene-1 (NDRG1) is known as a metastasis suppressor. However, there are also reports that it may also have pro-oncogenic functions in the pathogenesis of breast cancer via the mTOR signaling pathway [178]. Similarly, high NDRG1 expression was correlated with tumor progression and brain metastasis in patients with aggressive breast cancer, and it was suggested that the upregulation of NDRG1 had worse clinical outcomes [179]. Despite the development of NDRG1 up-regulators (such as the di-2-pyridylketone thiosemicarbazone class) [178], further studies are required to clarify the exact role of this protein in breast cancer progression.

#### 5.4.6. Chimeric Antigen Receptor (CAR) T-Cell Immunotherapy

CAR T-cells are genetically engineered T-cells patients that express a synthetic receptor to bind to a tumor antigen. The FDA recently approved two CAR T-cell therapies against the C19 protein in the treatment of acute lymphoblastic leukemia and diffuse large B-cell lymphoma [180]. Despite dramatic responses in hematologic malignancies, the use of this personalized cellular therapy in solid tumors is in its early stages. TNBC is an attractive area of research for applications of CAR T-cell therapy. Folate receptor, MUC1, c-Met, integrin, HER2, ROR1, and TEM8 are among the targeted receptors in breast cancer in recent studies [181]. Several recent publications have reviewed the advancements of this approach in breast cancer [182,183,184,185,186,187].

## 6. Conclusions

While molecularly targeting breast cancer has come a long way in the last two decades, we are still heavily relying on chemotherapeutic agents. Even in combinatorial approaches, molecularly targeted drugs are often combined with chemotherapy (Table 1). On the other hand, many of the clinical trials in recent years have not delivered the promising results that were expected. Since we assume these clinical trials were pursued based on promising results in vitro and in pre-clinical studies, we can speculate that the heterogeneity of the cells in every patient (let alone the heterogeneity among different patients included in each study) may be a contributing factor. Among molecular targets in breast cancer, hormone-related targets are still the center of attention. This seems logical since ER-positive breast cancers are the majority of breast cancers diagnosed. However, in addition to the fact that TNBC cases would not benefit from this approach, downstream effectors of ER and PR could also be triggered by other mechanisms (Figure 1). This means that even if the drug works perfectly and completely inhibits the molecular target, the targeted mechanism could potentially still be functional.

One of the extensively studied areas in molecular targets is the crosstalk between apparently different signaling “pathways” (e.g., crosstalk between the estrogen receptor pathway and major signaling pathways in cancer cells; Figure 1). These crosstalks make it possible for the cancer cells to find alternative pathways to overcome the inhibition of their molecular targets. Common downstream effectors such as CDKs (Figure 2) or mTOR (Figure 5), as well as upstream effectors such as EGFR (Figure 1), the HER family of receptors (Figure 2), Src (Figure 4), or RTKs (Figure 5) are examples of points of contact between the “pathways” and molecules that can affect multiple signaling pathways. Considering these crosstalks and the concepts of vertical (combining upstream and downstream targets in the same pathway) vs. horizontal (combining targets from parallel pathways) could potentially increase the impact of the combinatorial approaches.

## Figures and Tables

**Table 2:** Summary of ongoing or completed clinical trials involving immune checkpoint inhibitors in breast cancer.

Drug	Target	Indication	Combination (Target)	Clinical Trials	Citation
Avelumab	PD-L1	HER2-Positive or TNBC	- Palbociclib (CDK4/6) Trastuzumab (HER2) and Utomilumab (4-1BB/CD137) Fulvestrant (ER) Talazoparib (PARP) Binimetinib (MEK1/2) TRX518 (GITR)	NCT01772004 (Phase Ib)	[71]
				NCT04841148 (Phase II)	-
	
				NCT03414658 (Phase II)	-
				NCT03147287 (Phase II)	-
				NCT03330405 (Phase II)	-
				NCT03971409 (Phase II)	-
				NCT03861403 (Phase I/II)	-
Durvalumab	PD-L1	HER2-Negative, HER-2-Positive, or TNBC	Tremelimumab (CTLA-4)	Multiple	[72,73]
			Olaparib (PARP)	Multiple	[73,74,75]
			Bevacizumab (VEGF)	NCT02802098 (Phase I)	[76]
			Cediranib (VEGFR)	NCT02484404 (Phase I/II)	[77,78]
			Trastuzumab (HER2)	Multiple	[79]
			Ibrutinib (BTK)	NCT02401048 (Phase I/II)	[80]
Camrelizumab	PD-1	TNBC	Apatinib (VEGFR)	NCT03394287 (Phase II)	[81]
				NCT04303741 (Phase II)	[82]
			Fuzuloparib (PARP)	NCT03945604 (Phase Ib)	[83]
			Famitinib (VEGFR)	NCT04129996 (Phase II)	[84]
Nivolumab	PD-1	HR-Positive, HER2-Negative, or TNBC	Ipilimumab (CTLA-4)	NCT02834013 (Phase II)	[85,86]
				NCT03409198 (Phase IIb)	[87]
			Bevacizumab (VEGF)	WJOG9917B	[88]
			Palbociclib (CDK4/6)/Anastrozole (aromatase)	NCT04075604 (Phase II)	-
Spartalizumab	PD-1	TNBC	LAG525 (LAG3)	NCT02460224 (Phase I/II)	[69]
				NCT03499899 (Phase II)	-
Cemiplimab	PD-1	Advanced breast cancer	SNS-101 (VISTA)	NCT05864144 (Phase I/II)	-
Sintilimab	PD-1	HER2-Positive or TNBC	Apatinib (VEGFR)	NCT04722718 (Phase II)	-
			Bevacizumab (VEGF)	NCT05386524 (Phase II)	-
			Anlotinib (Multiple RTKS, including VEGFR)	NCT04877821 (Phase II)	-
			Trastuzumab/Pertuzumab (HER2)	NCT05429684 (Phase III)	-
Ipilimumab	CTLA-4	HER2-Negative breast cancer	Nivolumab (PD-1)	NCT02834013 (Phase II) NCT03409198 (Phase IIb)	[85,86] [87]
Tremelimumab	CTLA-4	Metastatic breast cancer	Durvalumab (PD-L1)	Multiple	[72,73]
LAG525 (Ieramilimab)	LAG3	TNBC	Spartalizumab (PD-1)	NCT02460224 (Phase I/II)	[69]
				NCT03499899 (Phase II)	-
Sabatolimab	TIM-3	Advanced breast cancer	Spartalizumab (PD-1)	NCT02608268 (Phase I/Ib)	[70]

BTK: Bruton’s Tyrosine Kinase; CITR: Glucocorticoid-Induced TNFR-Related protein; VEGF: Vascular Endothelial Growth Factor; VEGFR: Vascular Endothelial Growth Factor Receptor; VISTA: V-domain Ig Suppressor of T cell Activation.

**Table 3:** Recent clinical trials involving targeting PI3K/AKT pathway (mostly in TNBC or HER2-positive breast cancer).

Drug	Targeted Protein	Indication	Used in Combination	Clinical Trials
Inavolisib	PI3Kα	HER2-Postivie	Pertuzumab/trastuzumab and Endocrine Therapy	NCT05306041 (Phase II)
Copanlisib	Pan-PI3K *	HER2-Positive	Pertuzumab and trastuzumab	NCT04108858 (Phase II)
Buparlisib (BKM120)	Pan-class I PI3K	HER2-Positive	Trastuzumab and paclitaxel	NCT01816594 (Phase II)
Eganelisib (IPI-549)	PI3Kγ	TNBC	Atezolizumab (PD-L1 mAb) and nab-Paclitaxel	NCT03961698 (Phase II)
Bimiralisib (PQR309)	PIEK/mTORC1/2 [119]	TNBC and HER2-negative	Eribulin (Microtubule Targeting Agent)	NCT02723877 (Phase II)
Panitumumab	EGFR	TNBC	Carboplatin and gemcitabine	NCT00894504 (Phase II)
Ipatasertib	AKT	TNBC or Hormone Receptor-Positive, HER2-Negative	Paclitaxel	NCT03337724 (Phase III)
Ipatasertib	AKT	TNBC	Atezolizumab and paclitaxel	NCT04177108 (Phase III)
Uprosertib (GSK2141795)	AKT	TNBC	Trametinib (MEK1/2 inhibitor)	NCT01964924 (Phase II)
Capivasertib (AZD5363)	AKT	TNBC	Olaparib	NCT02208375 (Phase II)
Rapamycin	mTORC1	HER2-Positive	Trastuzumab	NCT00411788 (Phase II)
Ridaforolimus	mTOR	HER2-Positive	Trastuzumab	NCT00736970 (Phase II)
Temsirolimus	mTOR	TNBC	Neratinib	
Vistusertib (AZD2014)	mTORC1/2	TNBC	Olaparib	NCT02208375 (Phase II)
Ribavirin	eIF4	Metastatic breast cancer	-	NCT01056757 (Phase II)

* With increased activity against PI3Kα and PI3Kδ [120]. ^‡^ The information included in this table was extracted from https://clinicaltrials.gov (updated 15 May 2023), limiting the searches to “Recruiting”, “Active, not recruiting”, or “Completed” Phase II, III, or IV studies.

**Table 4:** Ongoing and completed clinical trials on Src inhibitors alone or in combination with other drugs.

Drug	Indication	Used in Combination	Outcome	Clinical Trials
Dasatinib *	TNBC	-	Did not increase baseline membrane EGFR	NCT02720185 (Phase II)
Dasatinib *	Advanced ^†^ breast cancer	-	No progression-free patient after 16 weeks	NCT00546104 (Phase II)
Dasatinib *	HER2-Positive	Trastuzumab and paclitaxel	~80% of patient showed partial or complete response	NCT01306942 (Phase I/II)
Dasatinib *	Metastatic breast cancer	Selumetinib (AZD6244; MEK inhibitor)	Closed early for futility (one out of 30 patients had clinical benefit) [131]	NCT00780676 (Phase II)
Tirbanibulin (KX2-391) **	Breast cancer with prior taxanes therapy	Paclitaxel	-	NCT01764087 (Phase II)
Saracatinib	HR-Positive	Anastrozole	No significant positive impact for patients receiving saracatinib in addition to anastrozole	NCT01216176 (Phase I/II)
Saracatinib	Metastatic or advanced breast cancer	-	Out of 9 patients, 3 stable and 6 with progression of cancer	NCT00559507 (Phase II)
Saracatinib	Advanced solid tumors	AZD2171 (VEGFR inhibitor)	-	NCT00475956 (Phase I)
Bosutinib *	Advanced HER2-negative	Exemestane	Terminated (unfavorable risk/benefit ratio)	NCT00793546 (Phase II)
Bosutinib *	Advanced HER2-negative	Letrozole	Terminated (unfavorable risk/benefit ratio)	NCT00880009 (Phase II)
Bosutinib *	Advanced breast cancer	-	~40% progression-free survival and 26.4% overall survival	NCT00319254 (Phase II)
Bosutinib *	Metastatic/advanced breast cancer	Capecitabine	Terminated (unfavorable risk/benefit ratio)	NCT00959946 (Phase I/II)

* Dual inhibitor of Src and Bcr-Abl. ** Also a tubulin polymerization inhibitor. ^†^ Stage IV or inoperable stage III. ^‡^ The information included in this table was extracted from https://clinicaltrials.gov (updated 19 May 2023). Non-selective Src inhibitors are not included.

## Data Availability

No new data was created for this manuscript.
